# Items

**Published:** 1878-11

**Authors:** 


### Items.

The following circular letter has been issued by the Surgeon General of the United States Marine Hospital Service:

*To Physicians and others residing in the cities and towns visited by the yellow fever:*

Acting upon the advice of members of the American Public Health Association, the Surgeon General of the Marine Hospital Service has organized a commission to gather and record all important *facts* relating to the commencement and spread of the present epidemic of yellow fever, with the view of establishing truths upon which the theory and practice of prevention of future epidemics may rest.

The gentlemen composing the commission—Prof Bemiss, of New Orleans, Dr. Cochran, of Mobile, and Prof. Howard, of Baltimore,—have already commenced their work in New Orleans, and will visit the principal places in which the yellow fever has prevailed.

The cordial co-operation of all who have facts to communicate bearing upon the subject under investigation is earnestly solicited for the commission.

A preliminary report of the facts, gathered up to the 19th day of November next, will be presented to the American Public Health Association, which will convene on that day in the city of Richmond, to discuss the report, and to advise the best course to be pursued in concluding the labors of the commission.

It is not intended to terminate the investigation on the 19th of November, but it is desirable that those interested in public hygiene, in all parts of our country, should meet in council prior to the assembling of Congress, as it is generally believed that legislative action will be promptly taken in reference to preventing future epidemics. The agitation of the public mind upon this important subject is proper and commendable, but herein lies the peril of hasty or not fully matured legislation, and it is hoped that by the action proposed, Congress may have the benefit of the advice of the representative men of sanitary science.

The invitation of the officers and executive committee of the American Public Health Association, extended for the Richmond meeting, is to " medical and sanitary authorities and other citizens who seek to promote the public health." This will afford an opportunity for all who desire to advise in reference to the work in hand.

If the voluntary contributions of money for the expenses of the commission are sufficient, it is intended to add to the commission a sanitary engineer, a microscopist and a meteorologist.

The first step in the investigation is, as already stated, an examination into the causes of the commencement and spread of the epidemic, now being prosecuted by the commission.

The second step is the study of the natural history of the disease itself, which will be undertaken if the contributions of money warrant.

It is believed that the study of the treatment of the disease should not be added to the labors of the commission. The contributions of experience in this direction will doubtless be numerous, and may be properly left to the medical journals of the country, and to individual reports.

Very respectfully,

JNO. M. WOODWORTH,

*Surgeon General U. S. Marine Hospital Service.*

WASHINGTON CITY, Oct. 10, 1878.

---

THE ILLINOIS STATE MEDICAL REGISTER for 1878–9, Dr. D. W. Graham, Editor, has just been issued. In the accuracy of its compilations and the neatness of its typographical appearance it is all that could be desired.

